# A bio-inspired microstructure induced by slow injection moulding of cylindrical block copolymers†
†Electronic supplementary information (ESI) available: Azimuthal intensity profiles for *X*-axis scans (a, b and c) and *Y*-axis scans (d, e and f) and various sample thicknesses: 0.95 mm (a and d), 0.45 mm (b and e), 0.23 mm (c and f). The injection point was at *X* = 0 and *Y* = 0 and the injection rate was 7 × 10^–8^ m^3^ s^–1^. See DOI: 10.1039/c4sm00884g



**DOI:** 10.1039/c4sm00884g

**Published:** 2014-07-09

**Authors:** Joanna Stasiak, Jacob Brubert, Marta Serrani, Sukumaran Nair, Francesco de Gaetano, Maria Laura Costantino, Geoff D. Moggridge

**Affiliations:** a Department of Chemical Engineering and Biotechnology , University of Cambridge , Pembroke Street , Cambridge , CB2 3RA , UK . Email: gdm14@cam.ac.uk; b Freeman Hospital NHS , Freeman Road, High Heaton , Newcastle upon Tyne , NE7 7DN , UK; c Department of Chemistry , Materials and Chemical Engineering , Politecnico di Milano, Piazza Leonardo da Vinci 32 , 20133 Milan , Italy

## Abstract

It is well known that block copolymers with cylindrical morphology show alignment with shear, resulting in anisotropic mechanical properties. Here we show that well-ordered bi-directional orientation can be achieved in such materials by slow injection moulding. This results in a microstructure, and anisotropic mechanical properties, similar to many natural tissues, making this method attractive for engineering prosthetic fibrous tissues. An application of particular interest to us is prosthetic polymeric heart valve leaflets, mimicking the shape, microstructure and hence performance of the native valve. Anisotropic layers have been observed for cylinder-forming block copolymers centrally injected into thin circular discs. The skin layers exhibit orientation parallel to the flow direction, whilst the core layer shows perpendicularly oriented domains; the balance of skin to core layers can be controlled by processing parameters such as temperature and injection rate. Heart valve leaflets with a similar layered structure have been prepared by injection moulding. Numerical modelling demonstrates that such complex orientation can be explained and predicted by the balance of shear and extensional flow.

## Abstract 2

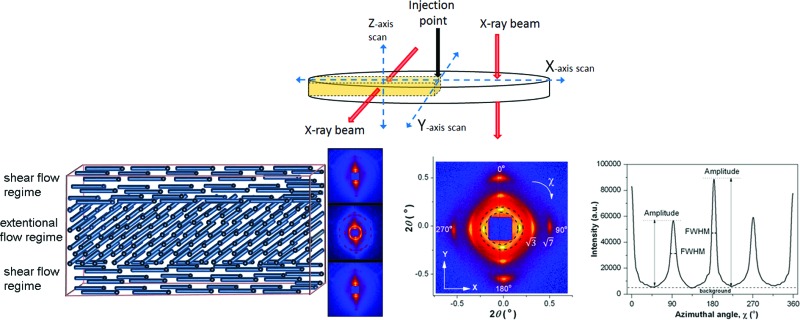
A bi-directional, layered microstructure in cylinder forming block copolymers results from the local balance of shear and extensional flow during slow injection moulding.

## Introduction

Block copolymers are of increasing interest because of their nano-scale morphologies, which can be utilized in a range of applications.^1–3^ Their properties often depend critically on molecular orientation induced during processing. One example is the properties of thermoplastic elastomers with a cylindrical morphology, where alignment of the cylindrical micro-domains results in orthotropic mechanical properties.^4,5^

It is well known that cylinder-forming block copolymers will orient strongly in the direction of flow when confined to a channel *e.g.* in a channel die or during extrusion.^6,7^ This results in strongly anisotropic mechanical properties, with (for the case of glassy cylinders in a rubbery matrix) a higher Young's modulus in the direction of orientation of the cylinders. We have recently shown that such behaviour can be extended to flow in two dimensions, by compression moulding between two parallel plates; the result is radially oriented cylindrical domains.^5^ It is therefore commonly assumed that orientation of anisotropic particles is governed by flow direction: thus when filling a mould one expects flow path induced alignment. However during morphological investigations of injection moulded films of poly(styrene-*block*-isoprene-*block*-styrene) containing 30 wt% styrene (SIS30), a block copolymer with cylindrical morphology, we unexpectedly found anisotropic domains forming a layered structure exhibiting bi-directional orientation. Using synchrotron X-ray diffraction, we have performed a detailed microstructural analysis, revealing distinct layers of orthogonal orientation at the skin and core of the samples. This bi-directional alignment was stable, extending throughout the sample. This complex micro-domain orientation can be explained by the balance of shear and extensional flow in different regions of the sample during the injection moulding process.

Such a layered structure with bi-directional orientation has not previously been reported in a solid material, although analogous bi-directional orientation zones have been observed in liquid crystalline systems subject to flow.^8–13^ Composite materials with a bi-directional microstructure could find a range of applications in the fabrication of functional devices. There is a general consensus that the formation of such structures can improve the biaxial tensile strength of materials, promoting high modulus and stiffness.^14–16^

Experimental measurements of orientation distribution and mechanical properties are combined with process modelling to explore the factors that control orientation. By linking the morphology to mechanical properties of the final solid material we are able to propose practical applications, such as the fabrication of prosthetic heart valve leaflets.

## Results and discussion

**Fig. 1:**
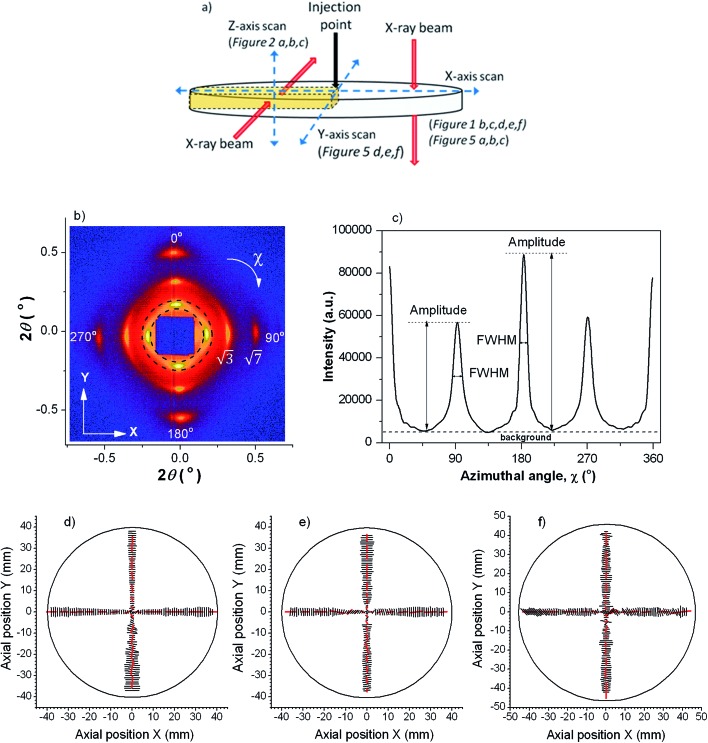
(a) Sample geometry with paths of SAXS scans and corresponding X-ray beam directions indicated. (b) Representative experimental SAXS pattern as scanned along the *X* axis; the area of azimuthal integration is indicated. Sample thickness was 0.95 mm and injection rate 7 × 10^–8^ m^3^ s^–1^. (c) Integrated azimuthal intensity profile. Peaks at 0° and 180° are from radially oriented cylinders; those at 90° and 270° are from circumferentially oriented cylinders. (d, e and f) Vector plot representations of molecular orientation along *X* and *Y* axes for various sample thicknesses: (d) 0.95 mm, (e) 0.45 mm, (f) 0.23 mm. Direction of the vectors indicates the orientation direction and the length of the vector is proportional to the degree of orientation.

Samples were injection moulded between two parallel plates to form discs of diameter 80 mm. The injection point was located at the centre of the top plate and the polymer melt was injected at 160 °C, as shown in Fig. 1a. Two injection rates and three sample thicknesses were investigated (see Methods section). The injection rates used were very gradual, at least 10 times slower than would be used in a typical industrial injection moulding process. The oriented structure induced by processing was observed after cooling.

Several different block copolymers have been investigated and all show qualitatively similar behaviour; for brevity and clarity, the results from a single material are focused on in this paper. In future it will be interesting also to consider the influence of injection temperature and cooling rate on the structures formed; our experimental set-up has limited our ability to do this, although we have found that small variations of the injection temperature have only small effects, which can be accounted for by changes in viscosity.

Microstructure and orientation was measured by Small Angle X-ray Scattering (SAXS) performed at the Diamond Light Source. To map the orientation distribution within the material, samples were X-ray scanned along the *X*, *Y* and *Z* axes as shown in Fig. 1a. X-ray patterns were recorded every 1 mm for *X* and *Y* scans; and every 0.1 mm for *Z*-axis scans.

Consider first X-ray patterns resulting from the X-ray beam passing through the sample in the *Z*-direction (*X* and *Y* scans). The SAXS images (Fig. 1b shows a typical pattern), contained a pair of meridional reflections (and associated meridional √3 and √7 reflections) and another pair of equatorial reflections (with associated equatorial √3 and √7 reflections); both pairs of reflections corresponding to a *d*-spacing of approximately 27 nm. The meridional reflections arise from cylindrical domains of the block copolymer oriented along the flow direction, whilst the equatorial reflections are from domains oriented perpendicular to the flow direction. The presence of √3 and √7 reflections confirm that the cylinders are arranged in a periodic hexagonal structure, as expected.^4,5^ The SAXS data were analyzed by azimuthal integration, as shown in Fig. 1b, for *χ* = 0–360°. The corresponding intensity profile in Fig. 1c shows four maxima at 0°, 90°, 180° and 270°, all peaks having similar integrated intensity (those at 0° and 180° are taller but narrower), indicating that similar amounts of radial and circumferential orientation were present in this sample.

Integrated azimuthal intensity profiles for full *X* and *Y* scans are provided as Supplementary Data. Bi-directional orientation is evident across the entire sample and for all three sample thicknesses. Azimuthal peak broadening at full width half maximum (FWHM_azimuthal_) was determined, as a measure of the degree of anisotropy for the two reflections at 90° and 180°.

Orientation angle for radial and circumferential alignment is represented in Fig. 1d–f as the angle of vector, while the vector's length is proportional to the reciprocal of FWHM_azimuthal_ for the relevant reflections. Significant variations of the degree of alignment and the angular positions of the four reflections were observed only in the close vicinity (5 mm or less for all sample thicknesses) of the injection point, associated with flow development after transition from the injection nozzle geometry to the mould. At larger radii, the degree of orientation in the radial and circumferential directions was high throughout the sample, and did not vary significantly with position. Because of the slow injection rates used, flow between the plates is laminar and so, once flow is fully developed (beyond the first few millimetres from the injection position), each point of the sample experiences a stable flow field in which strong orientation of anisotropic structures (such as cylinders) is to be expected, induced by shear or elongational forces. The direction of orientation at any point will depend on the details of the flow field at that point; because in our geometry shear (radial) and elongation (circumferential) are always perpendicular, we always observe either radial or circumferential orientation, but nothing intermediate.

**Fig. 2:**
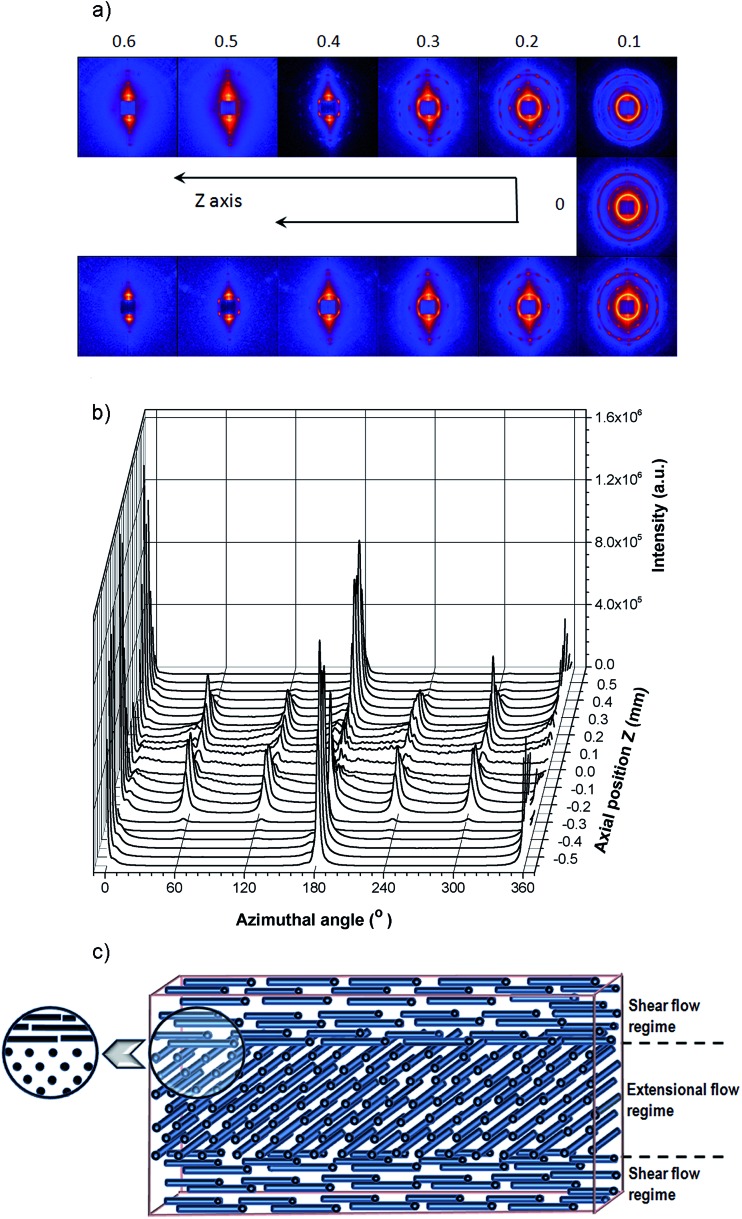
(a) SAXS images and (b) corresponding azimuthal intensity profiles, as a function of the sample depth (in the *Z*-direction). Sample thickness was 0.95 mm and injection rate 7 × 10^–8^ m^3^ s^–1^. (c) A radial cross-sectional schematic of the alignment of cylinders within the injection moulded sample (not to scale).

To probe the distribution of the two orthogonal orientations across the thickness of the material, we cut out a thin radial strip of the 0.95 mm thick sample and looked at its cross-section by performing a *Z*-axis SAXS scan. Representative X-ray images taken at incremental *Z* positions are shown in Fig. 2a. At the top of the sample the images showed two vertical reflections, indicating radial alignment (parallel to the flow direction) of the microstructure. Moving deeper into the sample the X-ray image changed to a hexagonal pattern, demonstrating close packed cylinders oriented circumferentially (perpendicular to the flow direction); it is interesting that a fully developed hexagonal pattern is observed, consistent with an almost single crystalline degree of organisation of the cylinders, at *Z* = 0.2–0.4 mm in Fig. 5. Close packed planes of this hexagonal structure were oriented horizontally (*XY*-plane) but not vertically. The hexagonal structure was slightly distorted by compression in the vertical (*Z*-direction) relative to the horizontal direction; for example the hexagonal pattern at *Z* = 0.2 mm has a *d*-spacing of 26.2 nm for the two meridional spots, and 29.2 nm for the four rotated by 60° to these. The middle zone (around 0.1 mm either side of *Z* = 0 in Fig. 5) contained an area in which the hexagonal spots of the layer just described are smeared into diffraction rings. This indicates that the cylinders were still oriented circumferentially and with a close packed hexagonal structure; however, in this region, micro-domains existed which were rotated relative to each other along an axis parallel to the cylinders.

The X-ray scattering observed below *Z* = 0 was symmetric to that above *Z* = 0, as expected.

As the sample was fairly thin compared to the size of the X-ray beam (250 μm vertically by 300 μm horizontally) each X-ray image represents the sum of morphological features present within the exposure area. As a consequence, in some images two meridional reflections characteristic of radial alignment overlay the hexagonal pattern arising from the circumferential orientation. This can be seen in some of the azimuthal intensity profiles of Fig. 2b, where 0° and 180° reflections characteristic of the radial skin layer overlay the 0° and 180° reflexions of the hexagonal pattern, giving a substantial increase of intensity for these two reflections of the hexagonal pattern.

In summary, the SAXS analysis shows that the material contained orthogonally aligned skin and core layers, as sketched in Fig. 2c.

By comparing the integrated X-ray intensities (from *X* and *Y* scans) corresponding to the radial and circumferential orientation, the fraction of each alignment present was estimated (summarised in Table 1) and found to be approximately constant across each sample. An increasing fraction of radial orientation was present for thinner samples and higher injection rates.

**Table 1:** Fraction of cylinders oriented radially, calculated from mechanical and X-ray data and numerical modelling (standard deviations of measurements shown in brackets)

Flow rate (m^3^ s^–1^)	Plate separation (mm)	Mechanical testing	X-ray analysis	Numerical modelling with a critical value of *ψ* = 30
2.00 × 10^–8^	0.97	0.33 (±0.06)	0.21 (±0.09)	0.34
2.00 × 10^–8^	0.44	0.56 (±0.04)	0.50 (±0.08)	0.57
2.80 × 10^–8^	0.30	0.76 (±0.04)	0.59 (±0.07)	0.68
7.00 × 10^–8^	0.95	0.48 (±0.05)	0.51 (±0.05)	0.37
7.00 × 10^–8^	0.45	0.67 (±0.05)	0.58 (±0.06)	0.59
7.00 × 10^–8^	0.23	0.86 (±0.10)	0.63 (±0.08)	0.76

**Fig. 3:**
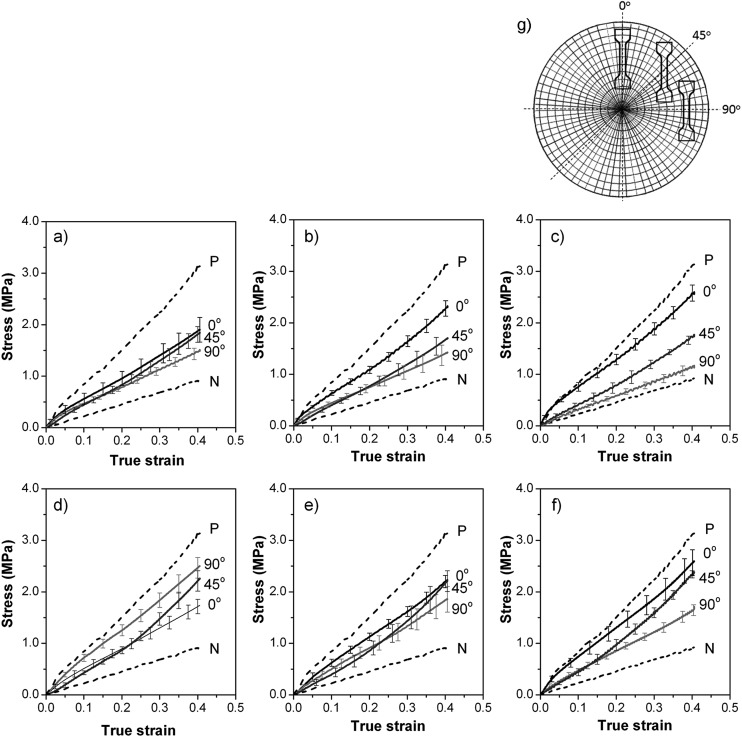
Stress–strain data for SIS30 injection moulded at 7 × 10^–8^ m^3^ s^–1^ into (a) 0.95 mm, (b) 0.45 mm, (c) 0.23 mm thick samples; at 2 × 10^–8^ m^3^ s^–1^ into (d) 0.97 mm, (e) 0.44 mm thick samples; and at 2.8 × 10^–8^ m^3^ s^–1^ into (f) a 0.30 mm thick sample. (g) Schematic of the layout of 0°, 45° and 90° specimens with reference to the disc's radius. P and N represent stress–strain data for unidirectionally oriented samples of SIS30 with cylinders aligned parallel (P) and normal (N) to the stretching direction, respectively.

The anisotropic mechanical properties of the samples were investigated by tensile testing. For comparison, samples having unidirectional orientation prepared by compression moulding in a channel die^4,5^ were also prepared and stretched parallel (P) and normal (N) to the cylinders' orientation. The tensile tests (Fig. 3) showed mechanical anisotropy of the tested samples, reflecting their microstructural anisotropy. To determine this mechanical anisotropy dog-bone shaped samples suitable for tensile testing were cut from the injection moulded discs parallel to the direction of flow (0° sample), perpendicular to it (90° sample), and half way between (45° sample), as shown in Fig. 3g. Tensile testing showed different mechanical properties for samples taken at different angles to the (radial) flow direction, as expected for the structural anisotropy measured by X-ray diffraction. Thus an injection moulded disc having cylinder orientation primarily in the radial direction (*e.g.* the 0.23 mm thick samples injected at 2 × 10^–8^ m^3^ s^–1^ shown in Fig. 3c) has a greater elastic modulus for the 0° sample than the 90° sample.

For the higher injection rate (Fig. 3a–c), the 0° samples were stronger than the diagonal (45°) followed by the 90° ones, indicating predominantly radial orientation. For the 0.23 mm (the thinnest) sample and the higher injection rate (Fig. 3c), the stress–strain curves for 0° and 90° directions approached those for unidirectionally aligned P and N, respectively, indicting almost exclusively radial orientation in this sample. The thinner the sample, with constant volumetric injection rate, the higher the flow rate between the plates; consequently the contribution of shear to microstructure orientation was dominant in the thinnest sample with the higher injection rate. Conversely, for the lower injection rate and largest space between plates, shear rate was low and so stretch was the dominant orientation mechanism; predominantly circumferential orientation resulted in the 90° direction being stiffer than 0° and 45° ones (Fig. 3d).

The fraction of the predominant orientation in each sample was estimated by comparison of its mechanical responses to those of uniaxially oriented samples P and N (see Tensile testing in Methods section). The results are shown in Table 1. If P and N samples are not in fact perfectly aligned, then this mechanical method of estimating orientation fraction will overestimate the degree of orientation for cases where there is a preponderance of radial or circumferential orientation. Except for the first row in Table 1, the mechanical method does give greater fractions of the dominant orientation than the X-ray method, where this differs significantly from 50%.

To obtain greater insight into the dynamic behaviour of the polymer during moulding, we performed computational fluid dynamics simulations, which allowed identification of the mechanisms responsible for the microstructural anisotropy. The flow system considered here cannot be solved analytically, due to its non-linearity with respect to radius, depth and viscosity. Numerical modelling of the system was carried out using ANSYS Polyflow.

Various sources have identified both shear and elongation as producing the forces necessary for orientation during flow of anisotropic morphologies in block copolymer systems.^17,18^ Elongation (stretch) of spatial elements in our flow geometry originates from the circumferential growth of fluid elements as they move from the centre to the outside of the discs (increasing radius). Shear forces are a result of creeping flow between narrow, non-slip plates. Thus, in this geometry, stretch and shear forces act orthogonally. We define the ratio of shear rate (*γ*′) to [biaxial] stretch rate (*ε*′) as the dimensionless group 
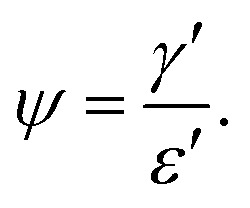
 We hypothesise that there exists a constant critical value of *ψ*, above which shear is dominant (resulting in radial orientation of cylinders), and below which stretch is dominant (resulting in circumferential orientation of cylinders).

**Fig. 4:**
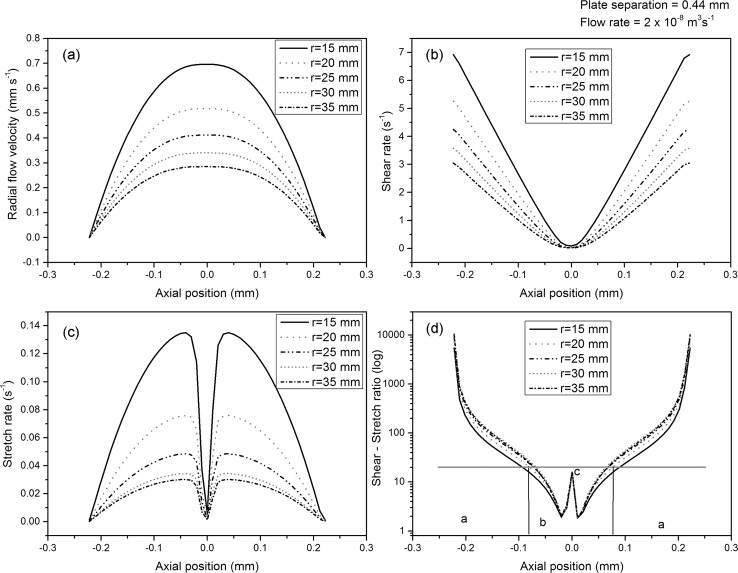
Numerical simulation results for an injection rate of 2 × 10^–8^ m^3^ s^–1^ and plate separation of 0.44 mm: (a) velocity profile, (b) shear rate profile, (c) stretch rate profile, (d) shear rate to stretch rate ratio profile, with distinct regimes indicated: (I and IV) where shear is dominant, (II) where stretch is dominant, and (III) a spike due to planar stretch, which may result in the rotationally disordered hexagonal structure observed around the axial centre of the sample. The horizontal line indicates the critical value identified.

A non-parabolic velocity profile develops between the thin plates, with a decrease in the maximum and average velocity as radius increases (illustrated in Fig. 4a for an injection rate of 2 × 10^–8^ m^3^ s^–1^ and plate separation of 0.44 mm), as required by mass conservation. The non-parabolic flow profile is indicative of the non-Newtonian behaviour of the fluid. The corresponding shear and stretch rates are shown in Fig. 4b and c, respectively. Fig. 4d shows the ratio of shear rate to stretch rate, *ψ*, believed to be the critical parameter in determining the orientation of cylinders.

It is interesting that although *ψ* varies across the depth of the sample it is almost constant at different radii; this explains why the orientations observed varied little with radius (see Fig. 1d), except close to the injection point. The high ratio of shear to stretch near the surfaces accounts for the radial orientation of cylinders here. Towards the axial centre of the sample, the ratio of radial shear to circumferential stretch decreases, resulting in circumferentially oriented cylinders. Exactly at the centre pure planar stretch occurs, whereas the stretch is biaxial in the non-central regions. Simple flows containing mixed stretch types have been observed by Baldawi and Wunsch.^19^ Planar stretch may be an inferior mechanism for cylinder orientation compared to biaxial stretch, and this may result in the rotationally disordered region seen in the SAXS patterns.

The modelling accounts for the effect of a change in injection rate. With decreasing flow rate, and plate spacing held constant, the proportion of radial orientation predicted decreases. Conversely, with reduced plate spacing, and overall flow rate held constant, so that the fluid velocity increases, a more parabolic flow profile results, with a decreased region in which circumferential stretch dominates, and thus more shear (radial) orientation. Consequently, we have a degree of control over the ratio of radial to circumferential orientation, by varying injection rate and sample thickness.

The ratio of radial to circumferential orientation calculated from the mechanical and X-ray data can be matched with reasonable accuracy by the modelling using a constant critical value of *ψ* = 30. At smaller values of *ψ* (<30) stretch is dominant in determining the orientation of cylinders, whilst shear is dominant for larger values. The fact that the critical value of *ψ* is substantially greater than 1 shows that stretch is a significantly more efficient mechanism of orientation than is shear. This is probably a consequence of shear resulting in strong rotational forces on an entrained cylinder, which are larger absent in elongational flow.

We observe good agreement between the measured and computationally predicted fractions of radial orientation (Table 1). The discrepancies between the data obtained by X-ray analysis and mechanical tests lie within two standard deviations; but may in part result from the assumption that P and N samples (used as standards for the mechanical tests) were perfectly, unidirectionally oriented.

Ongoing experimental studies show that the formation of bi-directional orientation also occurs, as a result of injection moulding, for other block copolymers with cylindrical morphology, such as poly(styrene-*block*-isoprene-*block*-butadiene-*block*-styrene) having 19 wt% styrene (SI-BS19) and poly(styrene-*block*-isobutylene-*block*-styrene) with 30 wt% styrene (SIBS30). All cylinder forming materials we have tested formed layers of orthogonally oriented cylinders similar to those described in detail in this paper for SIS30. Rheological differences result in different detailed patterns of orientation for different materials under the same processing conditions. These differences can be satisfactorily explained by modelling of the type described above, inputting only simple rheological measurements. It is thus possible to predict and control the microstructure formed by an interaction of the material being injected, the injection geometry and the processing conditions, including injection rate and temperature.

The ability to predict and control the formation of bi-directional orientation during processing is essential for engineering materials for specific applications, especially those requiring anisotropic mechanical performance. Since analogous bimodal microstructures exist in biological materials, it is believed that such structures are necessary to mimic the superior functional properties of natural tissues.

Native aortic heart valve tissue exhibits anisotropic material behaviour which is directly related to its microstructure. The valve leaflet consists of layers exhibiting highly anisotropic arrangements of collagen fibres. The fibrosa and ventricularis layers contain circumferentially oriented fibres, with the function of bearing stress during loading. There is also a layer of elastin present, oriented mainly radially in the ventricularis, and its function is to maintain a specific collagen fibre configuration and to return the fibres to their unloaded state intact when the load has been released.^20,21^ The mechanical anisotropy of human aortic heart valve leaflets is evident in measured values of the elastic modulus, which is much higher in the circumferential (14.5 MPa) than in the radial direction (1.5 MPa).^22^ Successful heart valve prostheses require the engineered material to meet and maintain demanding functional mechanical requirements, ideally in conformity with native valves. Numerical modelling has shown that even a small amount of orthotropy in the prosthetic material can significantly improve the mechanical behaviour of the valve, and that an appropriate orientation of the fibres can contribute to optimizing the stress distribution in the leaflets.^23^ Claiborne *et al.* have shown that block copolymers similar to those described in this paper are suitable for use in prosthetic heart valves and show low thrombogenicity, although they have not investigated the microstructural or mechanical anisotropy of the materials.^24–26^

**Fig. 5:**
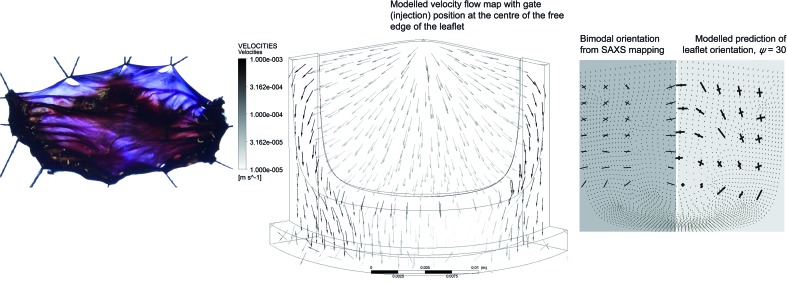
Bi-directional orientation in a porcine aortic valve (left); predicted flow pattern during injection moulding into a heart valve mould from the centre of each leaflet (centre); measured (by SAXS) and modelled bi-directional orientation in an injection moulded valve leaflet (right).

Fig. 5 shows the experimentally determined (by SAXS at I22, Diamond Light Source) orientation in a leaflet of an injection moulded heart valve made using SIS30 injected into a stainless steel mould *via* injection points at the centre of the top of each leaflet. Note that each heart valve took approximately 20 minutes to injection mould (compared to a few seconds for a typical industrial process); this gives an indication of how slow the injection process must be in order to achieve bi-directional orientation.

The calculated flow pattern in the valve and the resulting orientation predicted in a leaflet are also shown in Fig. 5, as is the fibrous structure of a native porcine aortic valve. The modelling of the development of orientation and mechanical properties during injection moulding in complex three dimensional geometries, such as a heart valve, is a little harder than in the two dimensional case (the flat disc) because the directions of shear and elongation need no longer be perpendicular. A development of the modelling for the 2D case (which will be described in detail elsewhere) allows us to predict orientation distribution and mechanical properties for complex 3D objects from the flow pattern in the mould. Fig. 5 shows an example of this for the heart valve geometry, but this can be extended to other geometries as required.

In addition to the injection geometry shown in Fig. 5, we have carried out experiments and calculations on injection along the whole top surface of each leaflet and at the two points at the extreme edge of the top of each leaflet. For brevity, these results are not shown, but resulted in very different orientation distributions in the manufactured leaflets. It is important that we are able to tune the orientation distribution achieved, using the processing conditions and injection geometry, and that we are able to achieve reasonable modelling of the flow pattern and orientation distribution in this complex, realistic geometry. Thus we will in future be able to optimise the experimentally achieved orientation distribution by an iterative process of modelling, measurement and mechanical testing. Of the injection geometries and processing conditions tested so far, the leaflet shown in Fig. 5 has an orientation distribution closest to that of the natural tissue, and so is expected to be an approximation to the prosthetic valve with the best mechanical properties; further development and refinement is, however, ongoing. For the heart valve application, tolerance without damage of many millions of repeated cycles (heart beats) of deformation is critical to its performance; fatigue testing is therefore essential. A prototype valve of the design shown in Fig. 5 is being tested *in vitro* in a pulse duplicator under quasi real time conditions (200 beats per minute; 120 mm Hg maximum pressure drop; 5 L min^–1^ flow rate) and has so far survived 3 million cycles without any detectable damage.

Another example of a native tissue with a bi-directional microstructure alignment is the outer coating of the eye, the cornea stroma. The cornea contains several overlapping layers composed of bundles of collagen fibrils surrounded by a soft matrix of glycoprotein. The collagen fibrils of 25–35 nm diameter run parallel to each other with fairly regular spacing, forming a layer (lamella).^27^ Lamellas about 200 μm thick each are crosswise stacked. The existence of parallel to the surface, but orthogonally to each other, oriented layers of collagen fibrils is responsible for the ability of the cornea to transmit light, while being mechanically resilient. Anisotropy in stromal architecture also results in mechanical anisotropy. It has been demonstrated that specimens stretched along the vertical direction of the cornea were up to 20% stronger than those stretched along the horizontal direction.^28^ It is believed that such microstructure allows maintenance of corneal strength and curvature.

## Conclusion

Although native biological tissues are more complex than the synthetic composite material examined here, not only in composition but also in their patterns of molecular architecture, the ability of cylinder-forming block copolymers to mimic the anisotropic structural and mechanical properties of these native tissues makes this group of materials attractive for engineering of bio-inspired systems. The case of particular interest for us is the development of improved prosthetic heart valves; anisotropic block copolymer leaflets can mimic the natural structure and improve the mechanical performance of the valve.^29–31^ Such polymeric valves have potential to show superior performance to the mechanical and bioprosthetic heart valves currently in clinical use. Our experimental observations, coordinated with numerical simulations, indicate that morphology and mechanical properties can be controlled by adjusting processing parameters such as mould geometry, temperature and injection rate.

## Experimental

### Materials

The block copolymer investigated was poly(styrene-*block*-isoprene-*block*-styrene) containing 30 wt% styrene, commercial name D1164P manufactured by Kraton Polymers, referred to as SIS30, having a weight average molecular mass of 131.2 kg mol^–1^ and a polydispersity index 1.11.

### Injection moulding

Injection moulding was at 160 °C, *via* a 1 mm diameter inlet pipe into the centre of two parallel circular plates of diameter 80 mm. The temperature dropped from 160 °C to 40 °C in approximately one minute, after the completion of injection. Two volumetric injection rates and three sample thicknesses were tested, as shown in Table 2.

**Table 2:** Investigated injection rates and sample thicknesses

Injection rate, m^3^ s^–1^	Sample thickness, mm
7 × 10^–8^	0.95 ± 0.05
	0.45 ± 0.02
	0.23 ± 0.03
2 × 10^–8^	0.97 ± 0.05
	0.44 ± 0.02
	0.30 ± 0.02

For the injection moulded heart valve shown in Fig. 5, injection was *via* a 1 mm diameter inlet pipe into the centre of the top of each leaflet; the injection rate was 6.2 mm^3^ s^–1^, with a barrel pressure 85.1 bar. The leaflets were cut out and analysed by SAXS on beamline I22 at Diamond. The thickness measured at several points of the leaflets was 0.35 ± 0.04 mm. This was modelled using a constant leaflet thickness of 0.35 mm and an inlet pressure of 75 bar, giving approximately the experimental injection rate in the modelled flow.

### X-ray analysis

Synchrotron Small Angle X-ray Scattering (SAXS) was performed on beamline I22 at Diamond Light Source, Harwell Science and Innovation Campus, UK. The energy used was 12.4 keV with a 6 m camera length and beamstop in the middle of the RAPID 2D detector. Further details of the beamline setup and technical characteristics can be found elsewhere.^32^

For quantitative analysis of the radial and circumferential orientation only the X-ray frames showing fully developed orientation, no closer than 15 mm from the injection point, were considered for the calculations. It was found that there was up to 10% variation in the characteristic *d*-spacing measured at various positions of the sample, for both radial and circumferential orientations. This indicates that individual elements of the fluid deformed during moulding and froze in a locally pre-strained state. Integrated peak intensity increases as *d*-spacing increases (due to the Lorentz polarisation factor) as a result of strain, for the same amount of material present in the X-ray beam. This has been quantified as a previously published stress – integrated peak intensity relationship for SIS30.^33^ Thus an intensity correction factor based on the observed *d*-spacing has been calculated and applied for each X-ray observation, to allow estimation of the proportion of radially and circumferentially oriented cylinders at each point in the sample.

Integrated azimuthal intensity profiles of *X* and *Y* scans for three sample thicknesses, are available as a figure in the Supplementary Material. This figure illustrates that bi-modal orientation is present over the whole of all three sample thicknesses examined.

### Tensile testing

The mechanical properties of block copolymers were investigated by uniaxial traction experiments, using a Texture Analyzer TA-TX2 from Stable Micro System, at 1 mm s^–1^ stretching and relaxation rate. By cutting out dog bone-shaped tensile bars at 0°, 45° and 90° with respect to the sample radius, as shown in Fig. 3g, three different angular arrangements of the microstructural orientation with respect to the stretching direction were measured. The initial length of samples after clamping was 10 mm and their width was 5 mm. The tensile tests were performed up to 0.5 strain. 8 samples of each orientation were measured for statistical purposes. In Fig. 3, true (Cauchy) stress (force divided by the actual cross sectional area of the sample) is plotted; and the strain is *ε* = Δ*L*/*L*_0_, where Δ*L* is the increase of sample length and *L*_0_ is the initial sample length.

Samples P and N were prepared by compression moulding in a channel die and had unidirectional orientation over the whole samples.^4,5^ Sample P was stretched in the direction parallel to the aligned cylinders; sample N was stretched in the direction normal to the aligned cylinders. The fraction of the predominant orientation in each sample was estimated by comparison of its elastic moduli at 0%, 10%, 20%, 30% and 40% strain to those of samples P and N. The arithmetic average of the moduli of samples P and N was matched to the moduli of the tested samples, giving the fraction of radial and circumferential orientation in the samples as the fractions of P and N required to match the experimental moduli. It is also possible to adopt this procedure in reverse, *i.e.* to predict the stress–strain curve for a particular sample from knowledge of the fraction of circumferential and radial orientation, by linear combination of the elastic moduli of P and N samples; we have made use of this to calculate stress and strain distribution in heart valve leaflets by finite element modelling (this will be reported in detail elsewhere).

### Modelling

Numerical modelling was carried out using ANSYS Polyflow. ANSYS solves the momentum and continuity equations at each finite element of a mesh. We assumed incompressible, steady, continuous, axisymmetric flow, with a purely viscous, 3-dimensional and isotropic medium. A no-slip condition was assumed at the interfaces with the upper and lower plates and the inlet wall. The plates were meshed with a radial resolution of 0.5 mm and axial resolution of 100 divisions between plates. The viscosity of the polymer was described by the Carreau–Yasuda equation, parameters (given below) being determined by rheometry experiments on the material (using an ARES parallel plate rheometer).

Rheological properties of SIS30 used in the Carreau–Yasuda equation are given in Table 3.

**Table 3:** Rheological properties of SIS30

*η*_∞_	5.16 × 10^3^ Pa s	Infinite shear viscosity
*η*_0_	1.62 × 10^5^ Pa s	Zero shear viscosity
*λ*	66 Pa s^–1^	Critical shear rate at which viscosity decreases
*a*	1.636	Width of transition region between zero shear and power law
*n*	0.145	Power law region exponent

Shear rate and stretch rate was derived from the deformation tensor, **D**.*ε*′= 6III_*D*_/II_*D*_ = 6 (det **D**)/(tr **DD**)where II_*D*_ and III_*D*_ are the 2nd and 3rd invariants of the deformation tensor, tr is the trace, and det is the determinant.
