# Supplementary figures and images for: A bio-inspired microstructure induced by slow injection moulding of cylindrical block copolymers
Source: Soft Matter. 2014 Jul 9;10(32):6077–86. doi: 10.1039/c4sm00884g (PMC4439735; doi:10.1039/c4sm00884g)

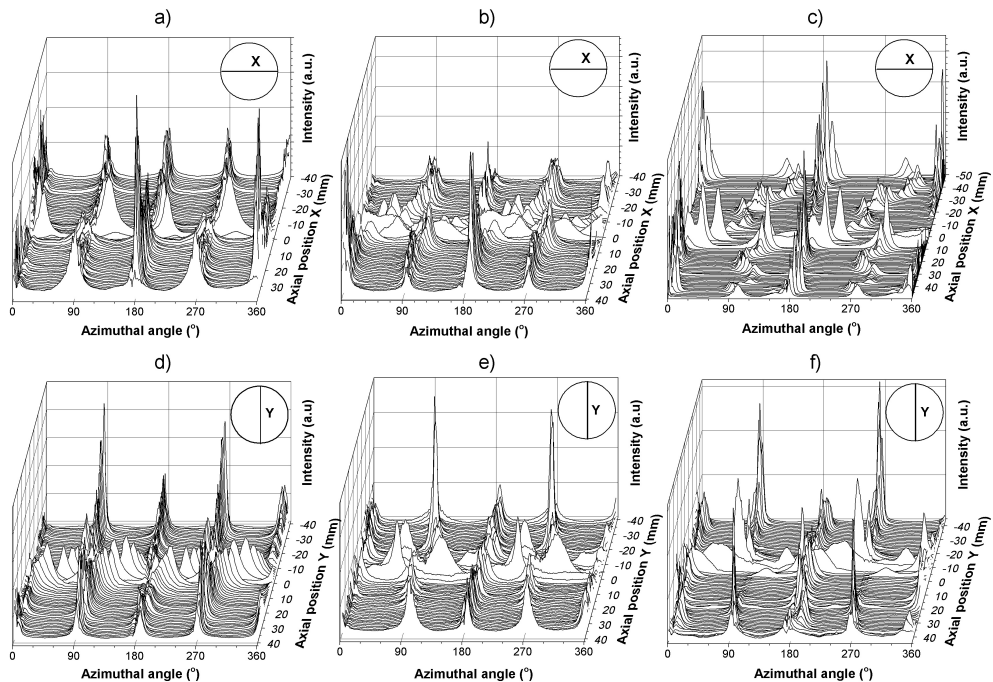

Supplement: Supplementary file 1 [file SM-010-C4SM00884G-s001.pdf]
